# Realization of a Pre-Sample Photonic-Based Free-Electron Modulator in Ultrafast Transmission Electron Microscopes

**DOI:** 10.1021/acsphotonics.5c00549

**Published:** 2025-10-08

**Authors:** Beatrice Matilde Ferrari, Cameron James Richard Duncan, Michael Yannai, Raphael Dahan, Paolo Rosi, Irene Ostroman, Maria Giulia Bravi, Arthur Niedermayr, Tom Lenkiewicz Abudi, Yuval Adiv, Tal Fishman, Sang Tae Park, Dan Masiel, Thomas Lagrange, Fabrizio Carbone, Vincenzo Grillo, F. Javier García de Abajo, Ido Kaminer, Giovanni Maria Vanacore

**Affiliations:** † LUMiNaD, Department of Materials Science, 9305 University of Milano-Bicocca, Milano 20126, Italy; ‡ LUMES, École Polytechnique Fédérale de Lausanne, Lausanne 1015, Switzerland; § Department of Electrical and Computer Engineering, Technion, Haifa 32000, Israel; ∥ CNR-Nano, Istituto di Nanoscienze Consiglio Nazionale delle Ricerche, Modena 41125, Italy; ⊥ IDES-JEOL, Akishima, Tokyo 196-8558, Japan; # ICFO-Institut de Ciences Fotoniques, 172281 The Barcelona Institute of Science and Technology, Castelldefels, Barcelona 08860, Spain; ¶ ICREA-Institució Catalana de Recerca i Estudis Avançats, Barcelona 08010, Spain

**Keywords:** ultrafast TEM, electron–photon interaction, electron-beam shaping, photonic electron modulator

## Abstract

Spatial and temporal light modulation is a well-established technology that enables dynamic shaping of the phase and amplitude of optical fields, significantly enhancing the resolution and sensitivity of imaging methods. Translating this capability to electron beams is highly desirable within the framework of a transmission electron microscope (TEM) to benefit from the nanometer spatial resolution of this instrument. In this work, we report on the experimental realization of a photonic-based free-electron modulator integrated into the column of two ultrafast TEMs for presample electron-beam shaping. Electron-photon interaction is employed to coherently modulate both the transverse and longitudinal components of the electron wave function (through lateral phase imprinting and temporal profiling, respectively), while leveraging dynamically controlled optical fields and tailored designs of the electron-laser-sample interaction geometry. Using energy- and momentum-resolved electron detection, we successfully reconstruct the shaped electron wave function at the TEM sample plane. These results demonstrate the ability to manipulate the electron wave function before probing the sample, paving the way for photonics-inspired imaging and spectroscopy techniques in ultrafast electron microscopy.

## Graphical abstract

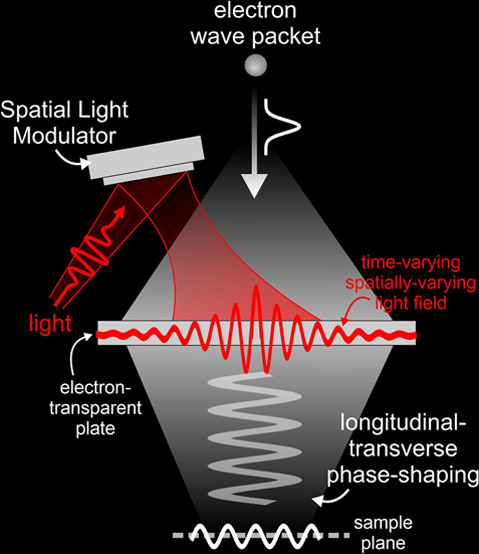

## Introduction

In recent years, electron–photon interaction (EPI) in ultrafast transmission electron microscopes (UTEMs) has been extensively adopted as a powerful tool for studying the ultrafast dynamics of laser-triggered nanoscale phenomena.^1–12^ Recently, the photonics community has shown growing interest in transferring advanced light-shaping techniques to the electron beam (e-beam) of UTEMs. In these experiments, preshaped laser fields transfer their modulation to the e-beam through EPI.^13,14^ Several approaches, such as optical parametric amplification (OPA),^15^ two-wave mixing,^16,23^ as well as dielectric laser acceleration (DLA),^17^ have been employed for longitudinal (energy-time) modulation of the electron wave packet, whereas spatial light modulators (SLMs),^18,19^ nanoconfined near fields,^20,21^ and Fabry-Pérot optical cavities^22^ have been used for transverse (momentum-space) shaping of the e-beam.

Two different approaches have been explored for e-beam modulation via light fields: elastic and inelastic interactions. Elastic EPI, mediated by the elastic ponderomotive force,^19,24^ offers the advantage of not requiring a physical interface to mediate the interaction. However, this method is constrained by the need for high laser intensities and is limited to phase-only modulation (unless two-color illumination is used to produce stimulated Compton scattering). In contrast, inelastic EPI has been successfully used to modulate both the phase and amplitude of e-beams. A first step in that direction has been taken by exploiting the photon-induced near-field electron microscopy (PINEM) approach^25–27^ for e-beam shaping.^20,21^ In PINEM, the interaction is mediated by the near field generated in the vicinity of a nanostructure (possibly supporting modes such as surface plasmon polaritons), allowing direct mapping of the structure’s complex optical field onto the transverse distribution of the electron wave function. While this correspondence is valuable for studying the near-field evolution, it limits the flexibility in e-beam shaping: predicting the final electron distribution requires numerical simulations to account for the near-field effects of the structure, limiting the range of patterns that can be imprinted on the e-beam.

To overcome these limitations, stimulated inverse transition radiation (ITR)^28–30^ has emerged as a versatile alternative form of EPI.^18,31–35^ Transition radiation (TR) consists of the electromagnetic waves emitted by an electron passing through an interface between two media with different refractive indices. Those waves are emitted to preserve the continuity of electromagnetic fields at the interface.^36^ In the inverse process (i.e., ITR), a stimulating electromagnetic field is already present, and the electron exchanges energy and momentum with such a field in a quantized manner (i.e., an integer number of photons). As a result, ITR allows for direct transfer of the laser phase onto the electron wave function without the near-field contribution present in PINEM, making the final e-beam profile effectively a Fourier transform of the SLM pattern used to shape the laser.^37^

Recently, Madan et al.^18^ demonstrated electron modulation mediated via inelastic EPI based on ITR using light preshaped by an SLM. In that work, the interaction was still occurring at the sample plane, where the film used to produce ITR was placed. Nevertheless, the authors foresaw a future technological implementation of a presample photonic-based free-electron modulator (PELM). This would be done by adding a new EPI interaction point in a presample stage along the TEM column. Different types of modulation can then be achieved by exploiting the multiplicity of phase patterns that can be imprinted on the SLM, as well as by replacing the SLM itself with other laser shaping technologies, such as optical cavities, DLAs, and OPAs, leveraging both spatially and temporally modulated light fields for controlling the e-beam in its multidimensional phase space before reaching the sample.

Realizing such possibilities will allow us to use preshaped e-beams to selectively probe nanomaterial dynamics with enhanced spatiotemporal resolution and sensitivity to specific properties and degrees of freedom. For instance, shaped electrons could be adopted to selectively probe low-frequency excitations in materials^35,38,39^ and to enable low-dose imaging of sensitive scatterers,^37^ as well as to enhance image resolution^24^ and increase contrast^15^ when interrogating materials showing very subtle changes, thus greatly expanding the capabilities of UTEMs.

Beyond these direct applications, the underlying approach also highlights a broader and increasingly important connection to the field of photonics. The ability to optically manipulate the wave function of free electrons is deeply rooted in photonic principles. In particular, tailoring the optical fields to interact effectively with free electrons often requires complex light shaping using nanophotonic structures.^40,41^ Shaped electrons are also promising probes of ultrafast optical phenomena with subcycle and even attosecond resolution^15,39,42^ and have recently been proposed for correcting aberrations in electron optics.^43^ Finally, beyond shaping, modulated electrons can sample and modify^44^ the statistics of optical fields, including the generation of quantum light states.

In this work, we present the experimental implementation of a PELM integrated into two UTEM systems: one at the University of Milano-Bicocca (UniMiB) and the other at the Israel Institute of Technology (Technion). Both setups were modified to position the PELM before the sample, although at different locations along the TEM column. At UniMiB, the PELM utilizes a SLM for transverse e-beam modulation, while at Technion, a noncollinear OPA (NOPA) is used for longitudinal modulation. By monitoring the electron wave function in its multidimensional phase space (energy, time, space, and momentum), we demonstrate both transverse and longitudinal presample modulation of the e-beam, driven by an externally controlled light field.

## Results and Discussion

### Electron-Photon Interaction and Coherence

As described above, our PELMs exploit ITR to facilitate EPI. In this process, electrons inelastically interact with a strong spectrally coherent light source, absorbing and emitting an integer number of photons.^27,44^ This quantized interaction imprints distinct peaks in the electron distribution, corresponding to multiples of the photon energy and momentum. For swift electrons (large energy compared with *ℏ*ω), the solution of the associated Schrödinger equation is simply given by the incident electron wave function multiplied by a temporal comb of period 2π/ω and a strength determined by the spatial Fourier transform of the traversed optical field at a spatial frequency ω/*v*, where *v* is the electron velocity. When decomposed in energy-momentum components, an electron comb emerges.^29–31,33,34,45^

To resolve these interaction peaks, and hence the quantized nature of EPI, the e-beam energy spread (and/or its momentum spread) must be narrower than the photon energy (or, respectively, than the photon momentum). Such narrow spreads are achieved when the e-beam longitudinal and/or transverse coherence lengths exceed the wavelength of the interacting photons.^46^ This is because the longitudinal and transverse coherence lengths directly determine the e-beam spread in energy and momentum, respectively, when averaging over the electron ensemble of wave functions.^47^ It is important to notice that, even when the coherence length is smaller than the photon wavelength although remaining of the same order of magnitude, it is still possible to detect EPI, with the only difference that the interaction peaks will be smeared out and the quantum nature of EPI hidden (note that this analysis covers only intrinsic coherence; extrinsic sources of broadening—such as mechanical or electrical instabilities—are separately discussed in the Supporting Information).

In the energy domain, EPI is detected if the temporal coherence of the e-beam is longer than or on the order of an optical cycle. This condition is easily achieved thanks to the small energy spread that results from the photoemission process at the cathode.^48^ As a consequence, the electron pulse has a temporal coherence ξ_*t*_ ≈ 5 – 10 fs, which is greater than the optical cycle (in our case, τ = λ/*c* ≈ 3.4 fs).^46^

In contrast, detecting EPI in the momentum domain is more challenging. In this case, the electron transverse coherence length, ξ_L_, must be greater than or on the order of the laser wavelength. In fact, the momentum coherence of the electron wavepacket (2π/ξ_L_) must be larger than the separation of fringes in the momentum space, *k*_L_, generated by the incident and reflected waves on the thin film of the optical modulator: *k*_L_ = (2π/λ)*f*_geo_, where *f*_geo_ is a geometric function (0 < *f*_geo_ < 1), while simultaneously the instrument has to be set with sufficient momentum resolution. Typical values of transverse coherence for thermionic electron sources, such as those used in our laboratories, are on the order of tens of nanometers,^49,50^ which is not enough for experiments with visible and infrared (IR) light. Therefore, specific microscope settings—namely, high dispersion diffraction—need to be used to increase the beam transverse coherence, as will be discussed below.

For e-beam shaping, it is crucial not only to achieve sufficient e-beam coherence but also to achieve coherent EPI, since preserving the phase information is critical for advanced imaging techniques. Coherent EPI occurs when the e-beam interacts with a homogeneous portion of the laser’s electric field. For longitudinally coherent EPI, the laser pulse is stretched in time to exceed the duration of the electron pulse.^31,33^ For transversely coherent EPI, the e-beam spot size is made smaller than the laser spot size.^33^

### Technical Implementation of the PELM Device

To realize EPI, we implement a pump–probe scheme. An IR laser pulse, produced by an Yb-based amplified femtosecond laser, is split in two branches. One pulse is up-converted to ultraviolet (UV) light by a fourth harmonic generation stage and is directed to the TEM cathode; its power is adjusted to generate single-electron pulses via photoemission. The other pulse is synchronized with the electron pulse and preshaped via the SLM. The two pulses interact via ITR on a light-reflective, electron-transparent metallic film at the PELM plane, where the structured laser field imprints its modulation onto the electron wave function. To maximize EPI, the shaped laser pulse is p-polarized with respect to the PELM film.^18^

The technical implementation of a PELM device requires integrating two presample access ports to the TEM column reaching the same inner position: an optical port for the modulating laser beam and a micromanipulator to adjust the stage holding the PELM film. This setup must satisfy two main design constraints: (i) sufficient lateral and vertical space within the column to accommodate the PELM film, and (ii) adequate transverse coherence of the e-beam to enable EPI detection. Here, we devise two configurations for integrating the PELM within UTEM setups, each with unique advantages and trade-offs, summarized in Table 1.

**Table 1:** Comparison of Pre-CL vs Post-CL PELM Configurations

feature	UniMiB (pre-CL)	Technion (post-CL)
PELM location	between C_0_ and C_1_–C_3_ lenses	after C_3_ lens
modulation type	transverse (SLM-shaped IR)	longitudinal (OPA-shaped IR)
optical access	single port (UV + IR)	separate UV and IR ports
e-beam coherence at PELM plane	moderate, via weakly excited C_0_ and e-beam clipping with PELM holder	high, via condenser lens system
e-beam manipulation between PELM and sample	possible, via condenser lens system	not possible
hardware modifications	additional column sections	minimal changes to HXA

Figure 1a shows the UTEM setup at UniMiB, with more details provided in the Supporting Information. This setup is based on a modified JEOL JEM-2100 TEM operating at 200 keV and equipped with a direct electron detector. The microscope column has been modified to accommodate two additional column sections before the C_1_–C_3_ condenser lenses (CLs). The upper section houses a supplementary condenser lens, labeled C_0_, while the lower section hosts: a single optical port for both UV and IR beams, an aluminum mirror to guide the UV beam toward the cathode, and the micromanipulator holding the PELM film (see Figure S1).

**Figure 1:**
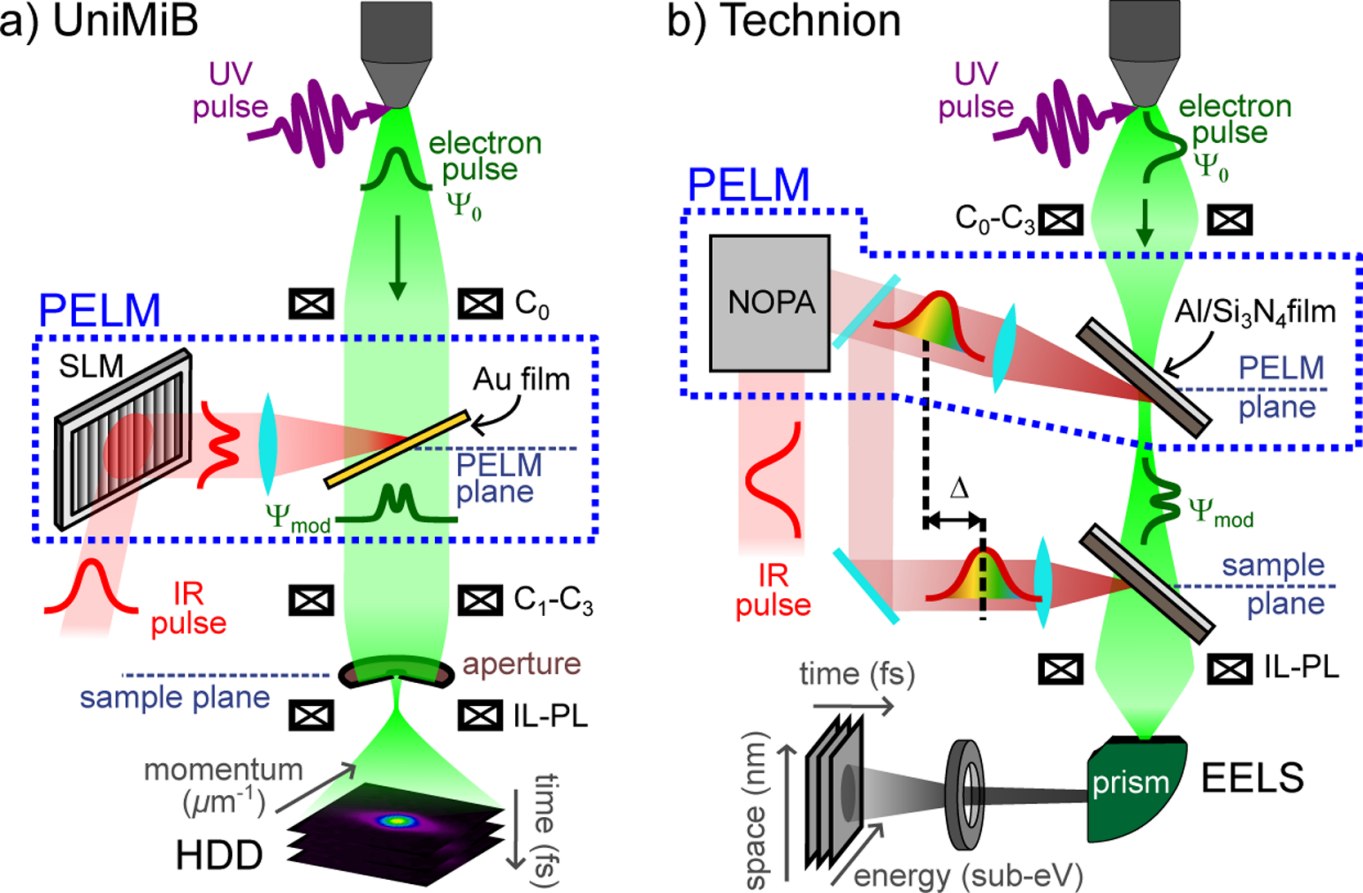
Schematics of experimental setups incorporating the photonic electron modulator (PELM) in two ultrafast TEM configurations at University of Milano-Bicocca (UniMiB) (a) and the Israel Institute of Technology (b). (a) Transverse electron-beam shaping at UniMiB. A spatial light modulator (SLM) is used to shape the transverse laser profile, which is then focused on a thin gold film positioned in a precondenser-lens (pre-CL) stage inside the TEM. Femtosecond electron pulses generated by UV laser pulses interact with the modulated IR laser pulses at the PELM-film surface via stimulated inverse transition radiation (ITR). A 20 μm aperture selectively samples a small portion of the modulated e-beam. High dispersion diffraction (HDD) patterns of the selected electrons are recorded. By scanning the aperture or laser, the entire transverse electron profile is reconstructed via the 4D-LSTEM method (see text for details). (b) Longitudinal electron-beam shaping at Technion. A noncollinear optical parametric amplifier (NOPA) is used to tune the optical cycle of the upper laser pulse, which is then directed onto a thin aluminum film deposited on a Si_3_N_4_ membrane located at the hard X-ray aperture (HXA) position, between the condenser and objective lenses. The electron pulses are modulated via ITR at the PELM stage. The longitudinal electron modulation is then probed via a double EPI scheme with an additional ITR interaction point at the sample plane.

Because of its pre-CL position, the PELM at UniMiB offers the key advantage of controlling the light-shaped e-beam via the CLs before reaching the sample. This pre-CL shaping is essential for implementing advanced imaging techniques that require, for instance, demagnification of the modulated e-beam at the sample.^37^ However, achieving optimal transverse coherence at this pre-CL stage is challenging; under typical imaging conditions, this is generally accomplished by demagnification with the CLs and transverse beam selection with the condenser aperture. Here, to reach the coherence required for resolving EPI at the pre-CL PELM plane, we substantially reduce the excitation voltage of the C_0_ lens (see Supporting Information) and we use the PELM stage itself as an aperture. Consequently, the e-beam diameter significantly exceeds that of the laser, resulting in substantial loss of useful electron flux, as many electrons fall outside the laser interaction region.

Figure 1b illustrates the UTEM setup at Technion^15^ (a real picture is also shown in Figure S6), based on a modified JEOL JEM-2100Plus TEM operating at 200 keV and equipped with a Gatan Image Filter and a K2 direct electron detector. This configuration includes a single additional column section with the C_0_ lens, the aluminum mirror for the UV beam, and the UV optical port. In this setup, the PELM film is accommodated by modifying the hard-X-ray aperture (HXA), located between the condenser and objective lenses. The IR-beam access to the PELM stage is provided through an optical port on the opposite side of the column, with an entrance angle of 20° relative to the horizontal plane.

At Technion, an optical pump line to the sample is implemented by further splitting the IR beam in two paths (see panel b of Figure S6 in Supporting Information). One path is directed to the sample plane via a zero-angle port, while the other enters the microscope at the PELM port, after passing through a fine-delay line.

With respect to the pre-CL configuration implemented at UniMiB, the advantages of the post-CL configuration at Technion are (i) a lower complexity in terms of technical design and practical realization, because the standard configuration of a TEM column is already designed to host a HXA; and (ii) a higher e-beam transverse coherence, which is ensured by the CL system. The latter aspect has been qualitatively investigated via electron trajectory calculations using the STEM-CELL software^51,52^ (see related section in the Supporting Information).

However, such post-CL PELM is limited by the tighter space available in this portion of the column, thus suffering from a lower flexibility in controlling the modulated e-beam before the sample and therefore limiting its versatility.

These two configurations highlight different engineering trade-offs in achieving structured electron-beam modulation within a TEM. Beyond the hardware-level considerations, the PELM approach itself presents intrinsic physical limitations that must be considered when designing experiments.

The device relies on inelastic scattering between electrons and a laser field, mediated by a thin material film. This interaction enables efficient modulation at relatively low laser powers (in the milliwatt range) unlike ponderomotive approaches that typically require much higher intensities.^16,19^ However, this benefit comes at the cost of reduced useable electron current, as a fraction of electrons is scattered away by the film. In addition, the interaction with the material may partially degrade the temporal and spatial coherence of the e-beam.^53^

Another source of loss arises from postselection: typically, only electrons that have interacted with light are considered in the final analysis, while unmodulated electrons are discarded. Despite these limitations, in the coherent EPI regime the interaction strength can be tuned such that the probability of zero-photon exchange is nearly zero.^31^

### Presample Electron Beam Shaping

Having discussed the technical design of the PELM devices, we now proceed to demonstrate their ability to control the e-beam properties within their multidimensional phase space.

### Transverse Modulation

Transverse modulation of the e-beam requires modifying both the spatial and momentum coordinates of the single-electron wave function. This can be achieved using spatially structured optical fields. In our case, we employ an SLM to shape the light field, but alternative approaches, such as nanoconfined near fields or photonic cavities, could serve a similar purpose.

At UniMiB, we achieve light-induced transverse e-beam modulation by exploiting inelastic EPI via ITR, which is mediated by a 5 nm thick gold film at the PELM plane. This thickness is not sufficient to approximate the PELM film with a perfect mirror, given that the skin depth of gold is around $t=\lambda /\left(2\pi \sqrt{\varepsilon_{2}/2}\right)\approx 27$ nm, where λ = 1030 nm is the wavelength of our laser. Using the approach outlined in the Supporting Information of ref 31, we simulated the EPI strength (β) as a function of the PELM film tilt angle α (as shown in the inset of Figure 2a), accounting for the nonideal mirror conditions. Our results, displayed in Figure 2a, indicate that an optimal interaction strength occurs at a tilt angle of α = −22°, which was therefore used in all following experiments.

**Figure 2:**
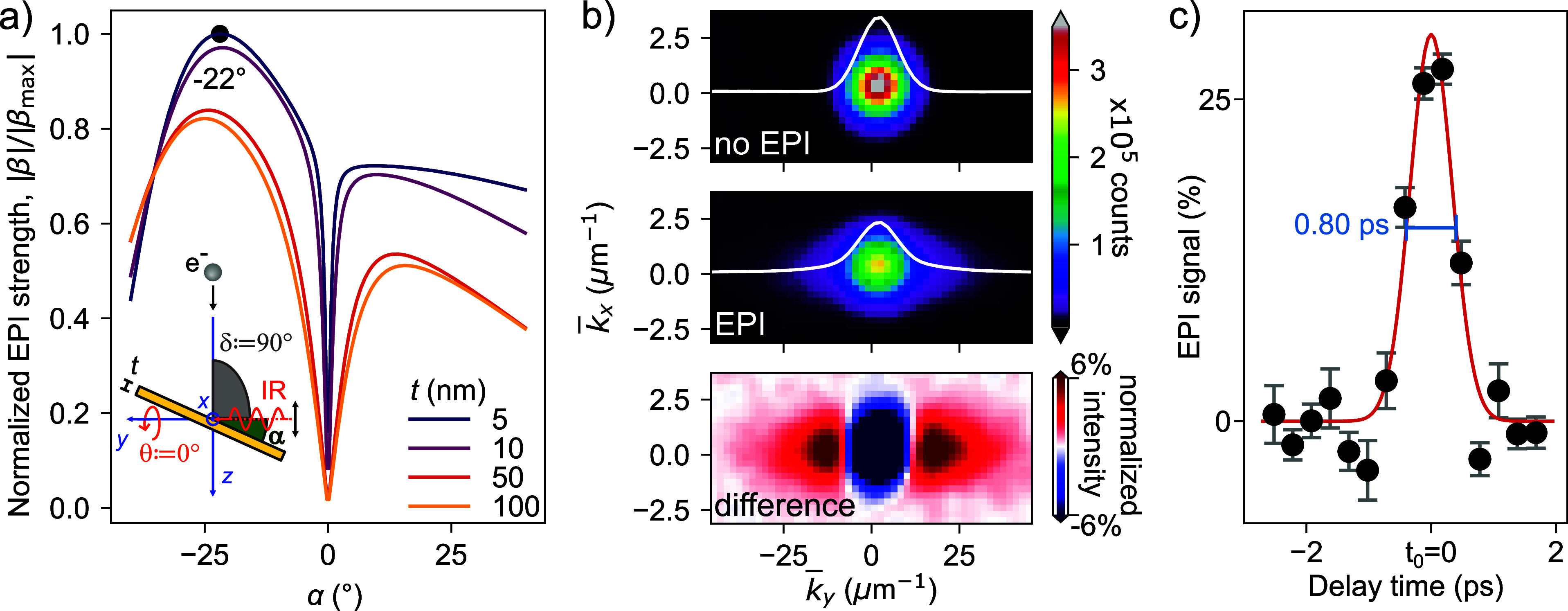
Analysis of EPI at UniMiB. (a) Simulated interaction strength as a function of the gold-film tilt angle α, accounting for nonperfect mirror conditions. The simulations are normalized to a maximum β_max_ = 0.8, extracted by comparing the simulated spectra with experimental measurements, as in the Supporting Information of ref 31. The maximum EPI strength occurs at α = −22°, which is used in all subsequent measurements. The inset illustrates the experimental geometry of EPI at the PELM film. (b) High dispersion diffraction (HDD) patterns of the e-beam under different conditions: without laser illumination (top panel), after interaction with IR photons (central panel), and their difference (lower panel). The interaction broadens the electron wave function along the *k*_*y*_ direction (laser propagation axis) and redistributes the intensity, reducing the signal near *k* = 0 and increasing it at higher *k*_*y*_. (c) Temporal evolution of the EPI signal (defined in the main text) as a function of the laser-electron pulse delay. Black dots are data and the red-solid line is a Gaussian fit. The extracted full width at half-maximum (fwhm) is 800 ± 30 fs. We define the temporal overlap *t*_0_ between the two pulses as the center of the Gaussian.

Here, we measure EPI by imaging the transverse momentum distribution of the e-beam in high dispersion diffraction (HDD) mode using a 100 m camera length on a direct electron detector (see Supporting Information for further information). The large camera length is crucial for detecting small momentum transfers. Indeed, the interaction imparts discrete multiples of the film-projected angular wave vector of light, $k_{L}=\left(2\pi /\lambda \right)\cos^{2}\left(\alpha \right)=\frac{2\pi }{1030\;nm}\cos^{2}\left(22^{{}^{\circ}}\right)\approx 5.2\;\mu m^{-1}$ (see also Supporting Information), which is equivalent to an angular deflection of θ ≈ (*k*_⊥_/*k*_∥_) = *k*_L_/(2π/λ_e_) ≈ 2.1 μrad, where λ_e_ ≈ 2.5 pm is the relativistic de Broglie wavelength for 200 keV electrons.

Figure 2b presents HDD momentum patterns of the e-beam under different conditions. In the absence of EPI (no light), the e-beam has a Gaussian momentum distribution (top panel). In the central panel, following interaction with IR photons, the electron distribution broadens along the *k*_*y*_-axis, corresponding to the laser propagation direction^31^ (see Supporting Information). From the top panel of Figure 2b, we extract the standard deviation σ_*k*_ = 5.45(1) μm^–1^ of the wave vector distribution, comparable to the expected sideband separation *k*_L_ ≈ 5.2 μm^–1^. As a result, the individual sidebands overlap, merging into the smooth profile presented in the central panel, despite the underlying quantized interaction. The lower panel in Figure 2b displays the difference between the upper two panels, enhancing the contrast to clearly highlight the effect of EPI. The interaction reduces the electron signal around *k* = 0 while increasing it at higher *k*_*y*_ values.

We performed the experiment as a function of the delay time between laser and electron pulse to achieve a precise temporal overlap between the two (*t*_0_: delay time = 0 ps). The results are shown in Figure 2c. The EPI signal is represented by the depletion of the direct e-beam and we quantify it as 1 – *A*_Voigt_, where *A*_Voigt_ is the amplitude of a Voigt function fitted to the normalized *k*_*y*_-integrated pattern. The temporal evolution of the EPI signal reveals a full width at half-maximum (fwhm) of 800 ± 30 fs, consistent with previous studies.^2^

Having demonstrated the ability to detect the effect of EPI on the electron wave function in momentum space at UniMiB, we performed 4D light-scanning TEM (4D-LSTEM) experiments to demonstrate transverse spatial modulation of the e-beam. In 4D-STEM, a focused e-beam is scanned across the sample while simultaneously capturing at each scan position a convergent-beam electron diffraction pattern, providing phase contrast analysis of the material under investigation.^54^ Here, a focused light beam is scanned across the e-beam on the PELM film while simultaneously capturing at each scan position an HDD electron diffraction pattern (see also Figure 1), providing transverse phase contrast analysis of the shaped e-beam. Therefore, in our case of 4D-LSTEM what in reality we scan across the sample is the portion of light-modulated electrons.

Figure 3 illustrates the experiments conducted with this 4D-LSTEM approach, with the electron and laser pulses in temporal overlap. A 20 μm aperture (as the ones commonly used in a TEM column) is placed in the sample holder, downstream of the PELM stage (see Figure 1). This aperture, smaller than the laser spot size, selects the electrons that interacted with a homogeneous portion of the electromagnetic field, condition needed for coherent interaction.^33^ Electrons passing through the most intense regions of the laser beam interact with more photons, resulting in a stronger EPI signal, and vice versa.

**Figure 3:**
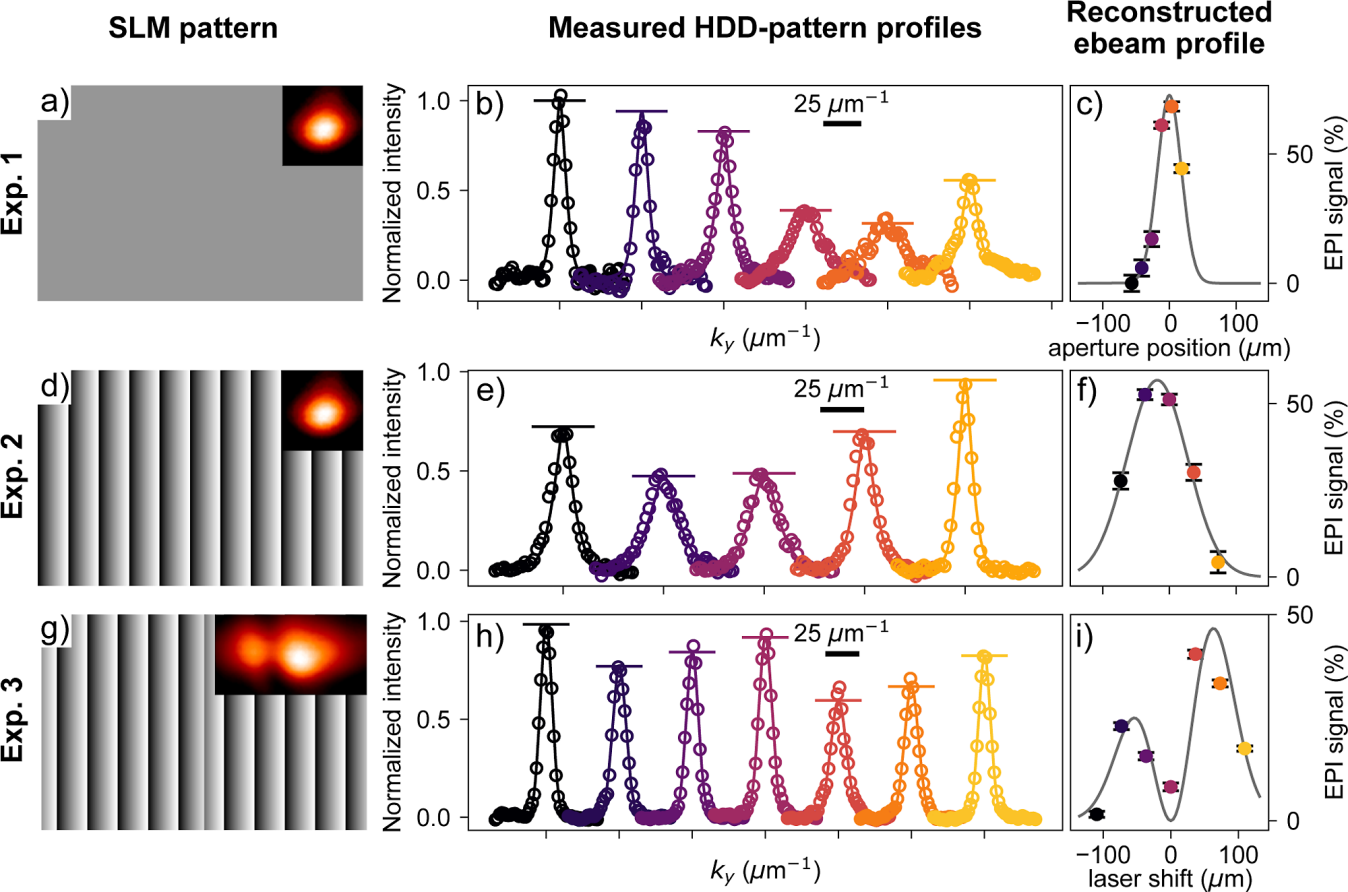
Reconstruction of electron-beam transverse profiles at UniMiB. (a,d,g) Patterns displayed by the SLM. The color scheme consist of a gray level of the pixels going from 0 to 255 to represent a laser phase shift from 0 to mod 2π. The insets show the modulated laser transverse profile recorded with a CCD camera in the conjugate plane (see Supporting Information for further details on the experimental setup). (b,e,h) *k*_*x*_-Integrated profiles (circles) of the HDD patterns acquired at maximum EPI. The colors represent different aperture positions (b) or laser shifts (e,h) coordinated with panels (c,f,i). The curves are laterally shifted for clarity. Solid curves are Voigt-function fits while horizontal lines represent the fitted amplitudes *A*_Voigt_. (c,f,i) Electron-photon interaction (EPI) signal, 1 – *A*_Voigt_, as a function of the aperture position (c) or the laser shift (f,i). Dot colors in these panels match the corresponding profiles in panels b,e,h. The gray line is a Gaussian fit (c,f) or a representative profile of the Hermite–Gaussian 01 mode (i).

The first row of Figure 3 depicts the initial experiment, in which the SLM serves as a simple mirror (pattern shown in panel a); the laser transverse profile is Gaussian, as shown in the inset. The aperture is scanned across this laser profile, and for each position, an HDD electron pattern is taken (as in Figure 2b) before and at *t*_0_ (see Figure 2c). The pre-*t*_0_ images serve as reference to normalize the *k*_*x*_-integrated profiles measured at maximum interaction. The resulting profiles, shown as circles in panel b, are fitted with a Voigt function (solid curves in panel b) and the fitted amplitudes are displayed as horizontal lines. Panel c shows the EPI signal, 1 – *A*_Voigt_, as a function of the aperture position, effectively reconstructing the e-beam profile at the aperture (sample) plane. We estimate the Gaussian-modulation diameter of the e-beam (at 1/*e* ^2^ intensity) to be approximately 72 ± 5 μm.

The second row of Figure 3 presents a similar experiment where the aperture remains fixed while the laser beam is scanned across the PELM film using the SLM. In this case, a blazed grating (shown in panel d) is employed to shift the laser beam in a controlled manner, based on the diffraction condition for the first order: Δδ = λΔ*n*/*H*. Here, Δδ is the laser tilt change, *H* is the SLM horizontal dimension and Δ*n* is the difference in the number of grating periods, which is varied to scan the laser on the PELM stage (see also Supporting Information). From these calculations, we can estimate the Gaussian modulation diameter at the PELM plane to have a 1/*e* ^2^-diameter of 180 ± 20 μm. By comparing panel f with panel c, we deduce that the e-beam has been demagnified by a factor 2.5 between the PELM and the sample plane, consistent with the almost-parallel configuration of the e-beam post-PELM (see Supporting Information) and verifying that experiment 1 and experiment 2 give the same result.

To further validate the transverse shaping capability of our setup, we performed a two-dimensional scan of the laser beam across the PELM plane. This experiment complements the one-dimensional modulation shown in Figure 4f by demonstrating control along both transverse directions. The resulting two-dimensional reconstruction of the e-beam modulation in the Supporting Information (Figure S5).

**Figure 4:**
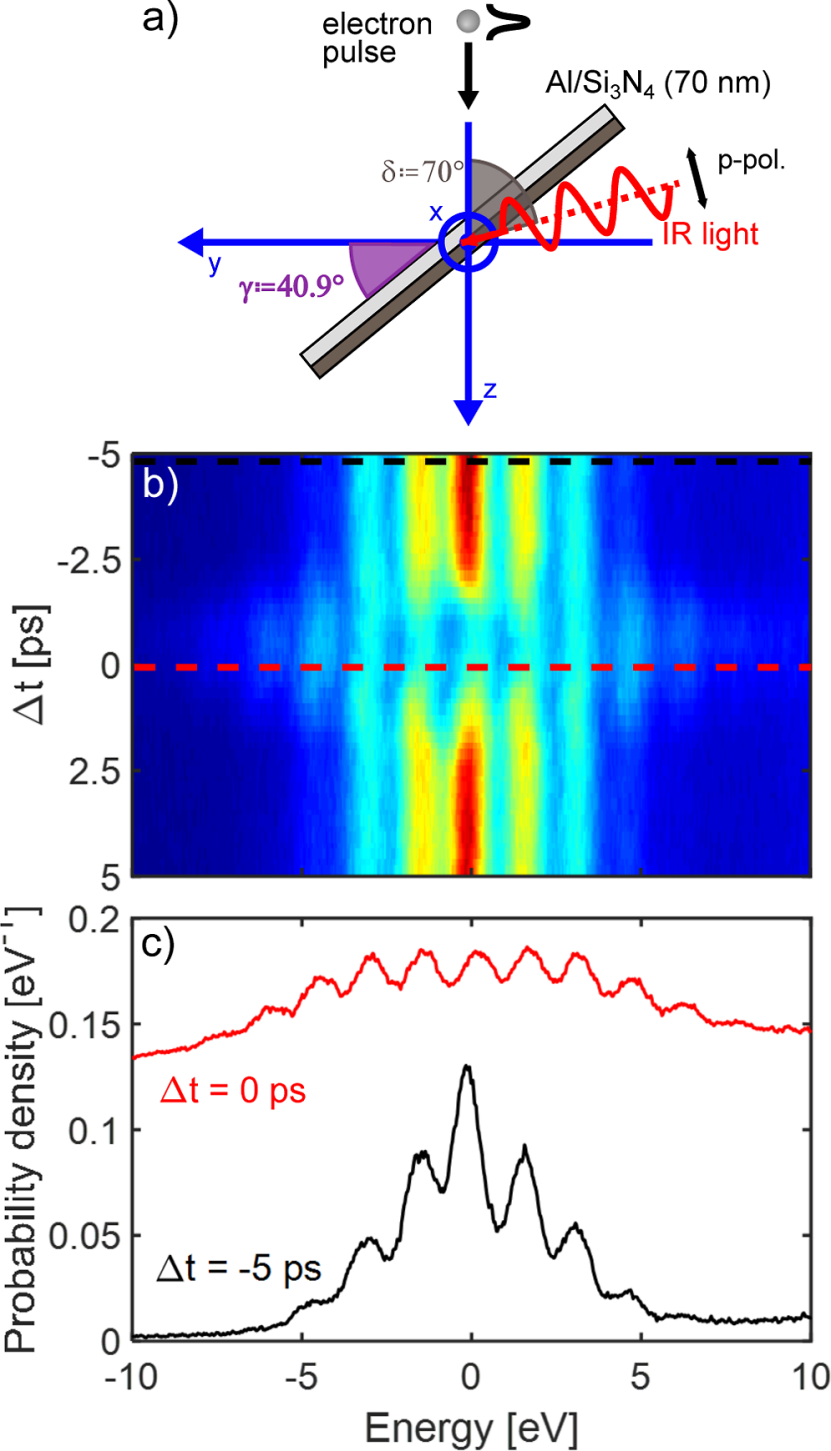
Analysis of double EPI at Technion. (a) Experimental geometry of EPI at the PELM film. (b) Electron energy spectra versus PELM time delay taken while the sample laser is at optimal temporal overlap, thus demonstrating *double* EPI. (c) Cross sections taken from the data in panel (a) along the color-coordinated dashed lines, showing the EPI spectra at Δ*t* = −5 ps (only sample EPI), and Δ*t* = 0 ps (both sample and PELM EPI).

The third row of Figure 3 depicts a further variation of the experiment, using a different SLM pattern. Here, a horizontal phase shift is superimposed on the blazed grating pattern (panel g), producing a two-lobed Hermite–Gaussian profile HG_01_ in the far field, as shown in the inset of panel g. The integrated HDD patterns in panel h allow reconstruction of the e-beam profile (panel i), which mirrors the two-lobed shape imparted by the light beam.

Our results clearly demonstrate the ability to manipulate the e-beam prior to sample interaction and to observe the resulting modulations at the sample plane. These findings demonstrate that such a PELM device is now ready for performing ultrafast measurements with a shaped e-beam on real samples. Furthermore, our system demonstrates versatility, not only in e-beam shaping but also in e-beam demagnification, as we have shown by reducing the e-beam pattern by a factor of 2.5, with further scalability possible.

### Longitudinal Modulation

Influencing the longitudinal phase of the e-beam requires temporal modification of its single-particle wave function. Because time modification beyond free evolution directly implies modification of the energy spectrum, we can access the electron longitudinal phase shift via direct imaging in the energy domain. From a technological point of view, appropriate time-dependent optical fields, as obtained via OPAs (used in this work), two-wave mixing, or DLAs, are needed in order to achieve the desired modulation.

At Technion, we achieve light-induced longitudinal e-beam modulation by exploiting inelastic EPI via ITR, which is mediated by a 30 nm-thick aluminum film deposited on a 40 nm Si_3_N_4_ membrane. In this case, the metallic film closely behaves as a perfect mirror.

For longitudinal modulation to be adopted in imaging, it is crucial that the light field imprints the same phase shift at every transverse position of the electron wave function. In order to ensure exact phase matching at the PELM plane between electrons and light interacting at the PELM-film interface, the film is tilted by an angle γ = 40.9° with respect to the horizontal plane (shown in Figure 4a). This angle can be obtained as tan­(γ) = sin δ/(*c*/*v* – cos δ), where δ is the angle between the electron and light (fixed at 70°), *c* is the light speed and *v* is the electron speed (*v* = 0.7*c* at 200 keV). Such expression is derived by imposing that the phase shift experienced by the electron pulse while interacting with the light field (ΔΦ_e_ = (ωΔ*z*/*v*) sin γ, where Δ*z* is the interaction distance), is equal to the projected phase of the light pulse on the PELM-film surface ( $\Delta \Phi_{L}=-\Delta z\frac{\omega }{c}\cos\left(\pi /2-\delta +\gamma \right)$ ). This condition makes sure that a spatially uniform longitudinal phase profile is imprinted on the electron wave function at the PELM plane.

Here, we measure EPI by monitoring the energy distribution of the e-beam via the acquisition of either energy-resolved spectra or energy-filtered images.

In Figures 4 and 5 we show the results of the simultaneous illumination of two Al/Si_3_N_4_ films, one placed at the PELM plane and tilted by γ = 40.9°, and the other one placed at the sample plane with a variable tilting angle. Such configuration results in two subsequent EPIs both governed by ITR mechanism.

**Figure 5:**
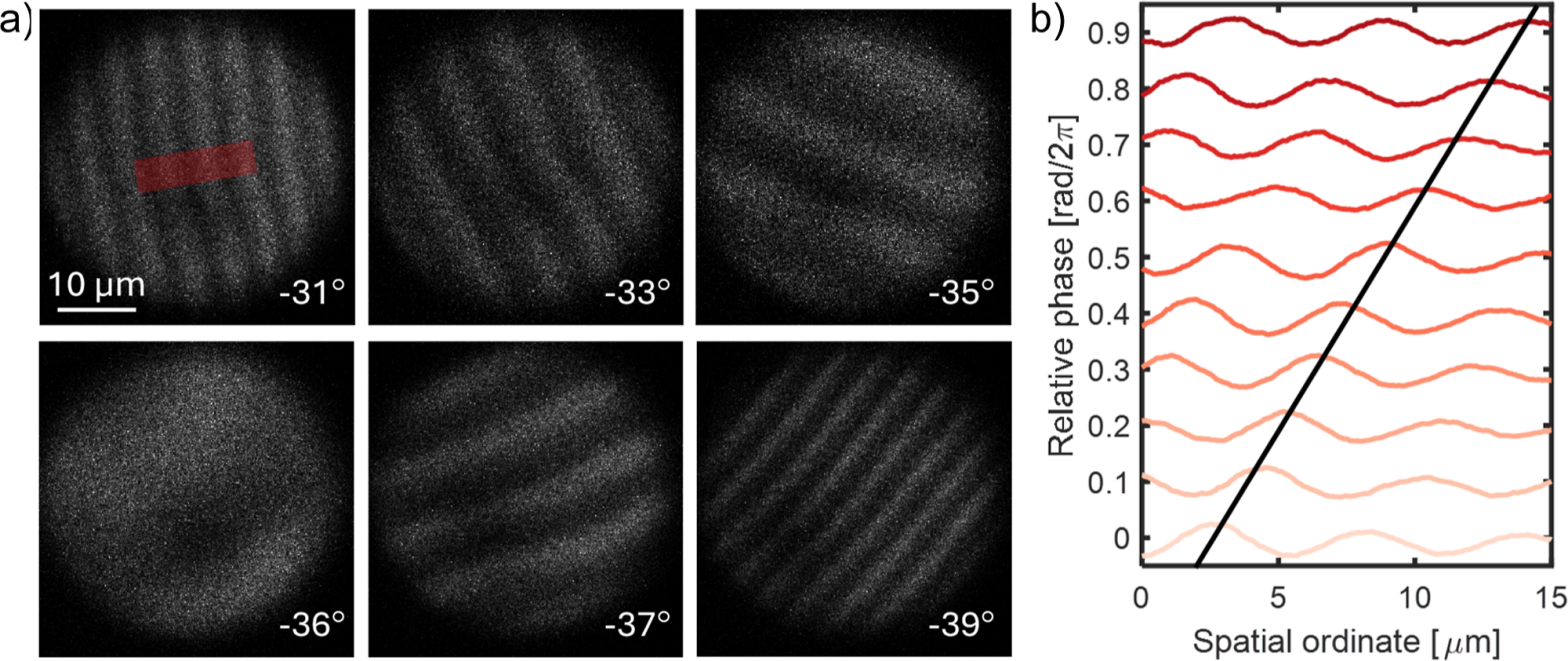
Electron interferometry and phase matching via double EPI at Technion. (a) Electron interferograms obtained by recording energy-filtered TEM images for different sample tilt angles, and at optimal temporal overlap at both the PELM and the sample. Sample tilt angle is indicated for each panel. The bottom-left panel (−36°) shows near-perfect phase matching (0 to 2π phase modulation across the ROI). (b) Cross sections of an interferogram, similar to those shown in panel (a), taken while sweeping the relative PELM-sample time-delay (or phase) across a full optical period (2.73 fs). The black line traces the position of one peak for varying delays. The red rectangle in the top-left panel of a illustrates the ROI used to draw the cross sections in (b).

In Figure 4b we observe the effect of such double interaction in the energy-time dimensions. Electron energy loss spectra are measured as a function of the delay time, Δ*t*, between the modulation pulse and the pump pulse, while keeping the electron pulse and the light pulse at the sample always at their optimal temporal overlap. At Δ*t* values very different from zero (black linecut in Figure 4c), the spectrum shows multiple quantized energy exchanges on both sides of the zero-loss peak (ZLP) as a result of EPI only at the sample stage. However, while approaching the optimal temporal overlap also between electron and light pulses at the PELM stage (red linecut), a stronger modulation across the spectrum is observed especially in energy regions far away from the ZLP.

A direct evidence of the coherent phase modulation experienced by the electron wave packet can be obtained via energy-filtering imaging of the spatial distribution of the e-beam following such double interaction. In Figure 5 we present the results of such imaging experiments, where energy-filtered images have been acquired as a function of the tilting angle of the sample film while maintaining optimal temporal overlap between electrons and light at both PELM and sample stages. Here, it is possible to observe the formation of a series of spatial fringes induced by the coherent superposition of the electron wave functions modulated by the two interaction points (PELM and sample). The periodicity of the fringes depends on the projection of the light wave vector on the tilted sample film and thus strongly changes when varying the sample tilting angle. The observed curvature of the fringes is associated with some local nonuniformity of the PELM membrane (apparent dark region and wiggle in the fringes in Figure 5a top-left and top-center). Also, in the data shown in Figure 5, an exposure time of 30 s per frame was used, and the total time to obtain the optical-cycle phase scan in Figure 5 is thus 5 min. Over these time-frames the microscope and optical setup were stable enough to support the data acquisition with the observed fringe visibility.

The short separation between the PELM position and the sample position is ideal for dispersive reshaping of the electron pulse to obtain attosecond longitudinal modulation of the beam in a Ramsey-like setup.^15,39,42^ In this respect it is crucial to have a precise correlation between the phase shift imprinted on the electron wave function and the relative phase difference between the two light pulses, the one driving the e-beam modulation and the other driving the sample excitation. In Figure 5a we show electron interferograms measured at a given sample tilting angle as a function of the delay time Δ*t* between the PELM laser and the pump (sample) laser within a single optical cycle. At each delay time, the measured shift of the electron fringes in the energy-filtered images is correlated with the optical phase difference (Figure 5b) as measured via a Mach–Zender interferometer. The latter is shown in details in Figure S6b of the Supporting Information: the laser beams directed at the PELM and sample planes are further separated and delayed with respect to each other before arriving on a CCD camera, where optical interference is recorded. From the measurements, we clearly observe a strong correlation between the optical phase and the electron phase, confirming that the PELM device is properly behaving.

## Conclusions

In conclusion, we have successfully integrated a PELM device into the UTEMs at UniMiB and Technion, enabling on-demand control over the electron wave function in both longitudinal and transverse directions. This was achieved through the integration of an additional EPI stage within the TEM column, allowing for e-beam modulation before the sample plane. This novel configuration expands the possibilities for using shaped e-beams in ultrafast electron microscopy experiments.

Given the capabilities of tailored e-beam shaping, PELM-equipped UTEMs open up new avenues for image-resolution enhancements, selective probing, and low-dose imaging. In particular, potential applications include single-pixel electron imaging, where a high number of patterns have to be imprinted on the electron wave function to obtain high-resolution images with minimal electron dose.^37^ Altogether, the integration of PELM into UTEM systems provides a practical and flexible foundation for future developments in phase-shaped electron microscopy.

## Supplementary Material



## Data Availability

B. M. Ferrari, C. J. R. Duncan, M. Yannai, R. Dahan, P. Rosi, I. Ostroman, M. G. Bravi, A. Niedermayr, T. L. Abudi, Y. Adiv, T. Fishman, S. T. Park, D. Masiel, T. Lagrange, F. Carbone, V. Grillo, F. J. García de Abajo, I. Kaminer, G. M. Vanacore, Realization of a Pre-Sample Photonic-based Free-Electron Modulator in Ultrafast Transmission Electron Microscopes, 2025, arXiv:2503.11313, arXiv preprint, 10.48550/arXiv.2503.11313 (accessed September 9, 2025).

## Notes

The Supporting Information provides detailed technical descriptions and geometry of the UTEM setup at the University of Milano-Bicocca, including the phase-modulating spatial light modulator. Calibration procedures for lens configurations (e.g., C_0_ and C_3_ lenses) and electron beam coherence optimization are explained, alongside calculations for camera length and momentum transfer during electron–photon interactions. The document also describes the role of apertures in controlling beam coherence and intensity, compares pre- and postcondenser lens PELM configurations, and discusses simulations using STEMCELL software to analyze electron beam properties. Additional setups, such as the Technion UTEM, and synchronization techniques for pump–probe experiments are briefly covered (PDF)
