# Supplementary material for: Realization of a Pre-Sample Photonic-Based Free-Electron Modulator in Ultrafast Transmission Electron Microscopes
Source: ACS Photonics. 2025 Oct 8;12(11):5864–73. doi: 10.1021/acsphotonics.5c00549 (PMC12643527; doi:10.1021/acsphotonics.5c00549)
Supplement: Supplementary file 1 [file ph5c00549_si_001.pdf]

Supporting Information: Realization of a Pre-Sample Photonic-based Free-Electron Modulator in Ultrafast Transmission Electron Microscopes

Beatrice Matilde Ferrari,[†,‡] Cameron James Richard Duncan,[†] Michael Yannai,[¶] Raphael Dahan,[¶] Paolo Rosi,[§] Irene Ostroman,[†] Maria Giulia Bravi,[†] Arthur Niedermayr,[¶] Tom Lenkiewicz Abudi,[¶] Yuval Adiv,[¶] Tal Fishman,[¶] Sang Tae Park,[‖] Dan Masiel,[‖] Thomas Lagrange,[‡] Fabrizio Carbone,[‡] Vincenzo Grillo,[§] F. Javier García de Abajo,[⊥,#] Ido Kaminer,[¶] and Giovanni Maria Vanacore[*,†]

†*LUMiNaD, Department of Materials Science, University of Milano-Bicocca, Milano, 20126, Italy*

‡*LUMES, École Polytechnique Fédérale de Lausanne, Lausanne, 1015, Switzerland*

¶*Department of Electrical and Computer Engineering, Technion, Haifa, 32000, Israel*

§*CNR-Nano, Istituto di Nanoscienze Consiglio Nazionale delle Ricerche, Modena, 41125, Italy*

‖*IDES-JEOL, Akishima, Tokyo, 196-8558, Japan*

⊥*ICFO-Institut de Ciences Fotoniques, The Barcelona Institute of Science and Technology, Castelldefels (Barcelona), 08860, Spain*

#*ICREA-Institució Catalana de Recerca i Estudis Avançats, Barcelona, 08010, Spain*

E-mail: giovanni.vanacore@unimib.it

UTEM setup at UniMib

Figure S1 shows the detailed layout of the Ultrafast Transmission Electron Microscope (UTEM) setup at UniMiB. The TEM used is a JEOL JEM-2100, equipped with both Bright-Field and Dark-Field STEM detectors. The femtosecond laser source is a Yb-based PHAROS laser from Light Conversion, and the Spatial Light Modulator (SLM) used for laser shaping is a HOLOEYE PLUTO, which modulates only the phase of the laser. The laser repetition rate for these experiments is 300 kHz and the power of the shaped IR beam is 10 mW. With a $1/e^2$ beam diameter of $80\,\mu$m, this gives an effective fluence at the sample $F_{\perp} \approx 9.7\,\mathrm{mJ/cm^2}$.

The setup also includes a Dectris QUADRO direct electron detector. The QUADRO features zero dark noise and high detective quantum efficiency at low electron flux, making it well suited for UTEM applications, where the photoelectron current is typically low. The high efficiency of the QUADRO enables high-quality imaging with short exposure times (of the order of tens of seconds in pulsed mode), which significantly mitigates the influence of extrinsic factors such as mechanical drift and electrical noise, which are key limitations in long-exposure ultrafast measurements. These instabilities are addressed past-acquisition through a robust frame alignment procedure based on the Enhanced Correlation Coefficient (ECC) algorithm implemented via the `cv2.findTransformECC()` function from the OpenCV Python library. This post-acquisition drift correction preserves the resolution over the entire acquisition period.

In the experiments presented in this paper, an aperture is positioned at the sample plane and two laser branches are in use: one UV branch for photo-generation of the probing electron beam and another branch, shaped by the SLM, to modulate the electron beam itself. The objective of this setup is to demonstrate that transverse electron beam shaping can be achieved at the sample plane.

The ultimate goal of our laboratory, however, extends beyond this preliminary experiment. Future works will place a real sample at the sample plane, and the shaped electron beam will be leveraged to selectively probe specific material excitations. To enable these

advanced pump-probe experiments, we are implementing a third laser branch to pump the sample. Optical access to the sample is provided via the Energy-Dispersive X-ray (EDX) port, which includes a leaded glass viewport.

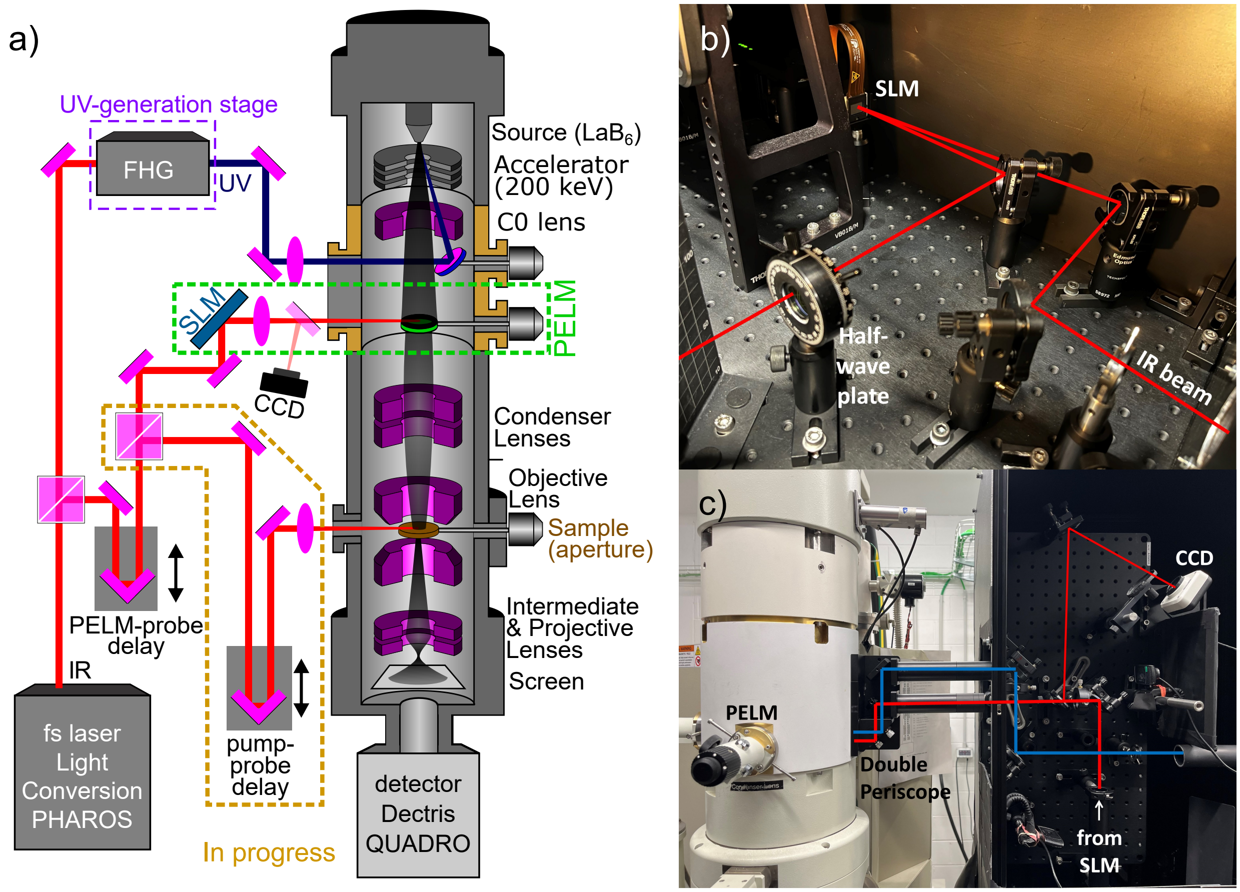

Figure S1: **Detailed layout of the Ultrafast Transmission Electron Microscope (UTEM) setup at UniMiB. a)** Schematics. Modified JEOL JEM-2100 TEM equipped with STEM and a Dectris QUADRO detector. A PHAROS femtosecond laser provides UV pulses for photo-generating electron beams and IR pulses shaped by a HOLOEYE PLUTO SLM for transverse electron-beam modulation. An aperture at the sample plane demonstrates shaping, with future plans to replace it with real samples for pump-probe experiments via a third laser branch introduced through the EDX port. **b,c)** Real pictures of our laboratory

Lens setup and calibration at UniMiB

Under typical operating conditions, the C_0 lens is excited slightly off its resonance voltage to maximize electron collection efficiency. The resonance voltage corresponds to the point at which the electron beam is optimally focused on the phosphor screen, as shown in Fig. S2. The figure shows the electron-beam current density on the phosphor screen as a function of C_0 lens excitation voltage, with the resonance voltage measured at 23.8 V. To achieve the large coherence needed to resolve Electron Photon Interaction (EPI), the C_0 lens is instead excited only 15 V.

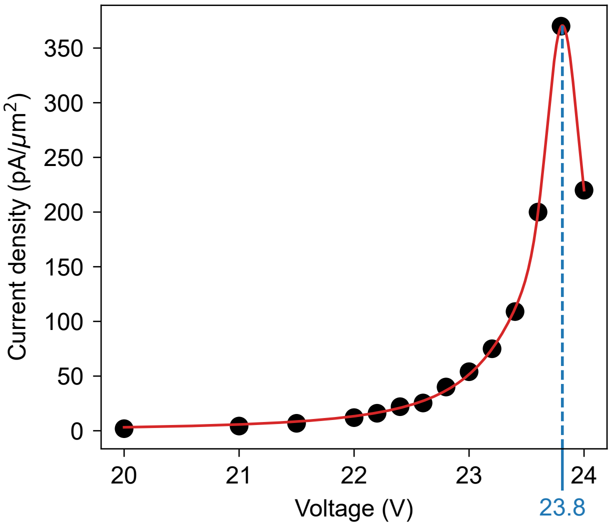

Figure S2: **Calibration of the C_0 lens at UniMiB. The electron-beam current density on the phosphor screen is shown as a function of C_0 lens excitation voltage, with the resonance voltage measured at 23.8 V.**

To directly correlate the electron profile between the sample plane and the PELM plane, we avoid forming crossovers between these two planes. This is achieved by operating the TEM in free lens control, turning off all lenses before the sample plane except for a weakly excited C_3 lens, which results in a minimally demagnified electron beam.

Operating the TEM in free lens control also allows us to reach the long Camera Length of 100 m through manual tuning of the intermediate lenses. To establish a momentum scale for our HDD patterns, we place a calibration sample, *latex spheres on diffraction grating*

replica (TED Pella product number 673), in the PELM plane. The calibration results are shown in Figure S3.

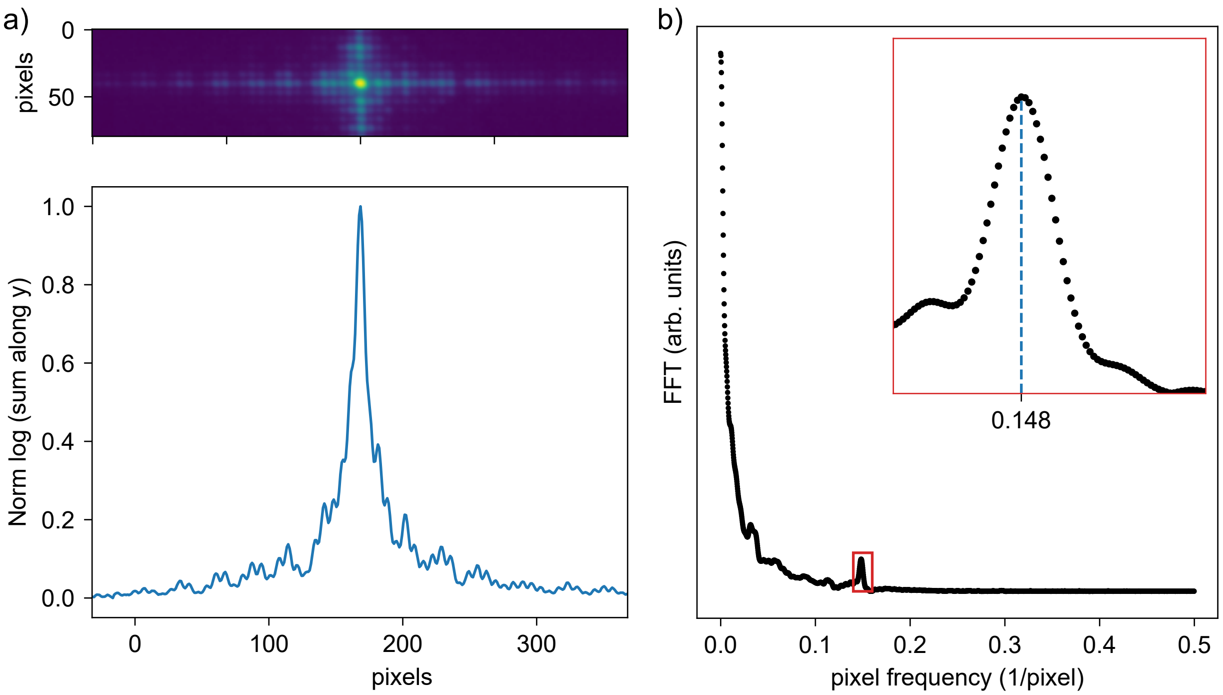

Figure S3: **Calibration of the momentum scale for High Dispersion Diffraction (HDD) patterns. a)** Diffraction pattern of the *latex spheres on diffraction grating replica* calibration sample placed at the PELM plane. The grating period is $2\,\mu\mathrm{m}^{-1}$. **b)** Fourier transform of the diffraction pattern, used to extract the peak period in pixels. From the calibration, we determine that one pixel corresponds to a reciprocal-space value of $0.296\,\mu\mathrm{m}^{-1}$, and the Camera Length is calculated to be $100\,\mathrm{m}$.

The reciprocal space sampling is then:[S1]

$$\Delta k = \frac{\text{grating frequency } [\mu\mathrm{m}^{-1}]}{\text{peak period [pixels]}} \times 2\pi[\mathrm{rad}] = \frac{2\,\mu\mathrm{m}^{-1}}{0.148} \times 2\pi\,\mathrm{rad} \approx 1.862\,\mathrm{rad}\,\mu\mathrm{m}^{-1} \qquad (1)$$

per pixel. The peak period, derived from the diffraction peaks in Figure S3a, is obtained from the Fourier transform shown in Figure S3b. Conventionally, the radians are dropped.

Using these measurements, we calculate the Camera Length as follows:

$$L_C = \frac{\text{peak period [pixels]} \times \text{pixel length [m]}}{\lambda_e \text{ [m]} \times \text{grating period [m}^{-1}\text{]}} = 100\,\text{m} \tag{2}$$

where $\lambda_e \approx 2.5\,\text{pm}$ is the de Broglie wavelength of relativistic electrons at 200 keV.

PELM-film geometry and momentum transfer at UniMiB

Figure S4 illustrates the experimental geometry used in our setup, following the layout and labeling convention in Fig. 1 of Ref.[S2] and Fig. S1 of Ref.[S3] for consistency and ease of comparison. The supplementary materials in these references provide derivations for the membrane-parallel components of the incident light wavevector that are transferred to the electrons during interaction.

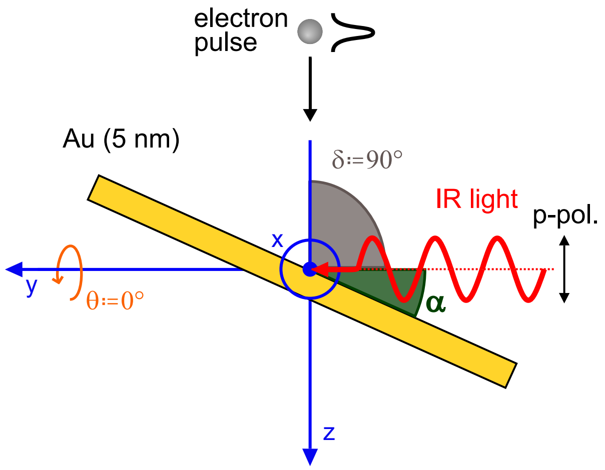

Figure S4: **Experimental geometry of electron-photon interaction (EPI) at the PELM-membrane**. The angles θ and α represent the membrane's rotation around the y-axis and x-axis, respectively, relative to the horizontal plane. The angle δ specifies the orientation between the electron beam and the light propagation direction. In our laboratory, $\theta = 0°$ and $\delta \approx 90°$ are fixed, while α is adjusted to optimize the interaction. The momentum transfer occurs predominantly along the y direction, aligned with the laser propagation.

In our configuration, the angles $\theta = 0°$ and $\delta = 90°$ are fixed (as shown in Fig. S4), while α is varied to optimize interaction. Using the formula from the supplementary information in Ref.,[S2,S3] we calculate the coordinates of the light-wavevector transfer components as:

$$q_{L\parallel}(x,y,z) = \left(0,\ \cos^2\alpha, -\frac{\sin 2\alpha}{2}\right) \tag{3}$$

The momentum transferred to the electrons is then given by $p_L = \frac{\omega}{c} q_{L\parallel}(x,y,z)$, where $\frac{\omega}{c}$ is the light wavenumber. As described in the main text, this momentum transfer occurs solely along the laser propagation direction y.

Spatial light modulator at UniMiB

The Spatial Light Modulator (SLM) used in this setup is the HOLOEYE Pluto 2.1, which consists of two primary components: (i) a micro-display and (ii) a driver unit. The micro-display comprises a 1920 x 1080 array of liquid crystal on silicon (LCoS) pixels, each measuring 8 μm $\times$ 8 μm. Each pixel can introduce a phase shift in the reflected light, with possible phase shifts ranging from 0 to 2π. This phase modulation is optimized for light wavelengths between 650 nm and 1100 nm, ideal for the laser's IR range. When the reflected light propagates through free space, the phase pattern generated by the SLM evolves into an intensity pattern. For optimal performance, the incident laser is set at a 7° angle to the SLM surface and is p-polarized.

The SLM driver unit refreshes the micro-display at a rate of 60 Hz and interfaces with a PC via an HDMI connection. This HDMI connection allows the driver to interpret image data sent from the PC as if the SLM were a standard computer display. Consequently, any software capable of displaying images on a computer monitor can, in principle, control the SLM's phase pattern. In our experimental setup, a custom Python-based program synchronizes the SLM with the Dectris QUADRO detector, the delay stage, and other components, allowing precise timing and control over beam shaping.

The SLM reflects an unchanged zero-order beam, making the first-order diffracted beam the primary component used for shaping. Consequently, the base pattern applied to the SLM is a blazed diffraction grating, which effectively separates the undesired zero-order

(unshaped) beam from the first-order (shaped) beam. When we refer to “using the SLM as a simple mirror”, we are actually describing this configuration, although technically it is not a simple reflection but a diffracted and directed first-order beam.

2-Dimensional reconstruction of the modulated e-beam at UniMiB

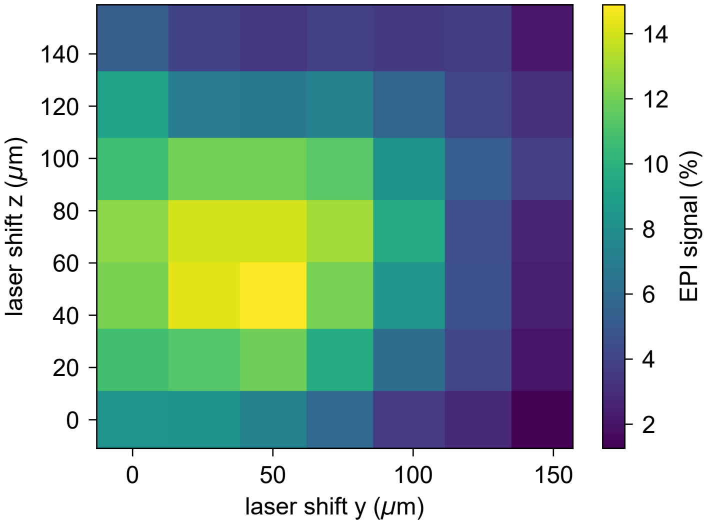

Figure S5: **Two-dimensional laser scan.** Pinem signal (see main text) as a function of the laser shift along y and z, as defined in Fig. S4.

In Fig. 4f of the main text, we show transverse Gaussian modulation of the electron beam along just one spatial coordinate. Nevertheless, we have performed a two-dimensional laser scan as well. This is shown in Fig. S5, further attesting to our ability of full transverse electron shaping.

UTEM setup at Technion

Figure S6 shows the optical setup at Technion UTEM, which is also described in detail in Ref.[S4] Independent laser paths deliver UV light to the TEM cathode and IR light to both

the PELM and sample planes. Two delay stages control the relative timing between these three beams, enabling synchronized pump-pump-probe experiments.

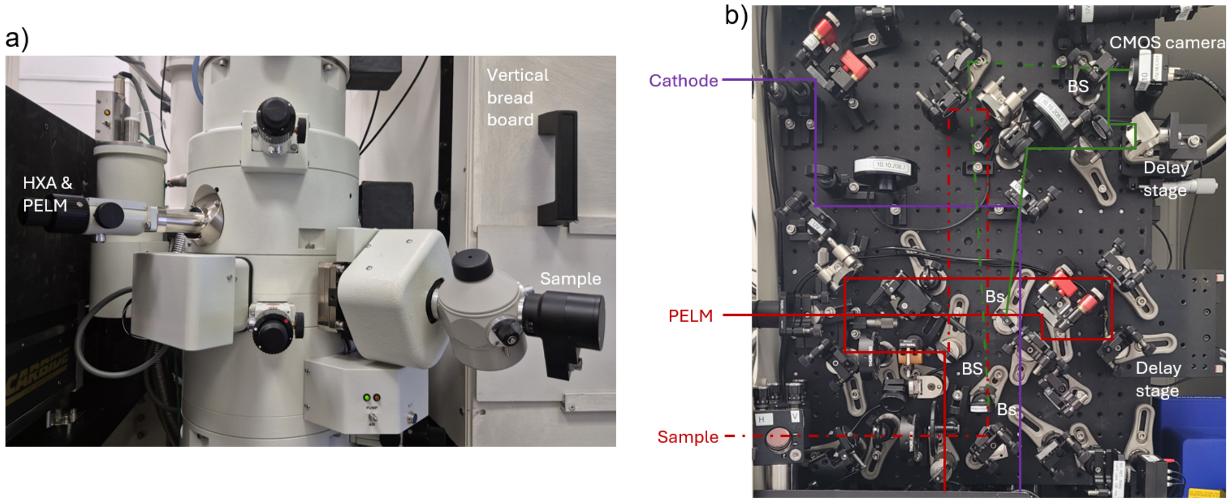

Figure S6: **Optical setup used at Technion UTEM. a)** Image of the microscope column showing the HXA mechanism connected to the PELM (inside the microscope column), the sample, and the vertical bread board (covered by an enclosure, see panel b). **b)** Detailed layout of the vertical bread board. Three optical access ports are installed on the column allowing the introduction of UV laser light to the TEM cathode (purple path), and adjustable wavelength laser to the PELM (red path) and sample (dot-dashed red path). A delay stage is introduced in the PELM beam path and is used to generate the relative delay between the sample and PELM beams. A small portion of the PELM and sample laser beams is sampled and used to measure their relative phase stability via an optical interferometer (green paths). BS – 50:50 Beam splitter, Bs – Beam sampler (90:10).

Simulation of the e-beam properties at the pre-CL and post-CL planes

In this section we will present electron trajectory calculations using the software STEM-CELL[S5,S6] for simulating the beam characteristics at the pre-CL stage and at the post-CL stage.

For PELM we need an interaction point that – by an electron optics point of view – plays the same role as one of the apertures. The different positions are shown in Fig. S7, which

represents the configuration of a JEOL microscope with the addition of a C0 lens and the drift sector.

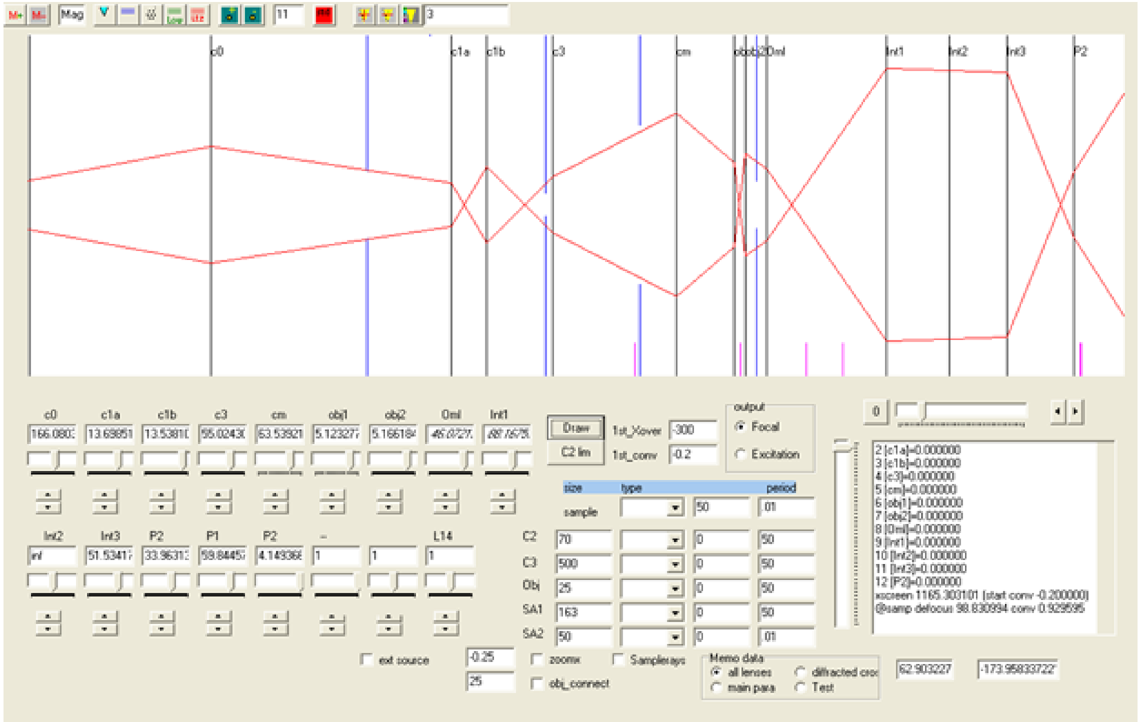

Figure S7: Lens diagram of a JEOL microscope: c0 is the additional condenser lens as discussed in the previous sectins; c1a, c1b and c3 form the condenser lens system; cm is the condenser minilens; obj is the objective lens that surrounds the sample; oml is the objective minilens; Int1, Int2 and Int3 are the intermediate lenses; p2 is the projective lens.

In the scheme, the first aperture is the pre-CL PELM interaction point (apertures are shown in blue in Fig. S7). The third aperture is the standard condenser aperture (CA). In between them, we marked also a fixed aperture of 1-mm size. The HXA aperture is also visible above the condenser minilens. The focal lengths and the lens excitation are that of standard operations with a condenser aperture of 100 μm; the beam size in the pre-CL PELM point is therefore around 100-200 μm.

As described in the main text, coherent transverse electron beam shaping would require, in principle, a large lateral coherence. In practice, the combination of Boersch effect and a large source size limits the lateral coherence of the electron beam, which is even more severe for a thermionic source in the case of pulsed emission. Nevertheless, one can always increase

the beam coherence but at the expense of the total intensity. Here, we discuss which of the two PELM positions is more favorable, considering in particular the ability to tune the coherence. In an ideal experiment we should be able to select the best trade-off between intensity and coherence and for some configuration this can be easier than for others.

The main reason for loss of lateral coherence is the finite extension of the source. For a given cathode and density of emission the only way to increase the coherence is by sacrificing beam intensity. The use of an aperture is able to cut away rays very far off the optical axis, consequently reducing the intensity.

The increase of the coherence can be thus seen by back propagating the rays from the aperture to the tip, thus realizing that we are observing only electrons from a selected part of the tip. The standard way to increase coherence is by using the condenser aperture CA, which determines also the convergence. However, there are cases in which this is not entirely true. Figure S8 and S9 below shows that the rays departing from two different points in the source end up in the same area in the CA plane, but in different points in the sample plane. In this specific case there is a negligible demagnification of the source to the sample. More precisely we considered a spherical wave produced by each point of the source and calculated which trajectories intercept the CA. In a more realistic model the emitted waves are very directional. Nevertheless, this model is qualitatively sufficient to describe the main effects.

The conclusion from the above simulations is that when using the CA as the only limiting aperture we have contribution from each part of the source that is imaged completely on the sample. There is an actual negligible demagnification of the source. While experimental conditions might improve beam characteristics due to the intrinsic directionality of emission, this setup lacks mechanisms for direct control over transverse coherence. One of the possible ways to actively control electron beam coherence is to couple two apertures in two sufficiently different planes. In the ideal case one could use a crossover to cut directly the external rays emitted from the side of the tip, but this is practically impossible as it would require a too small aperture. Still, one of the two apertures must be relatively close to the crossover

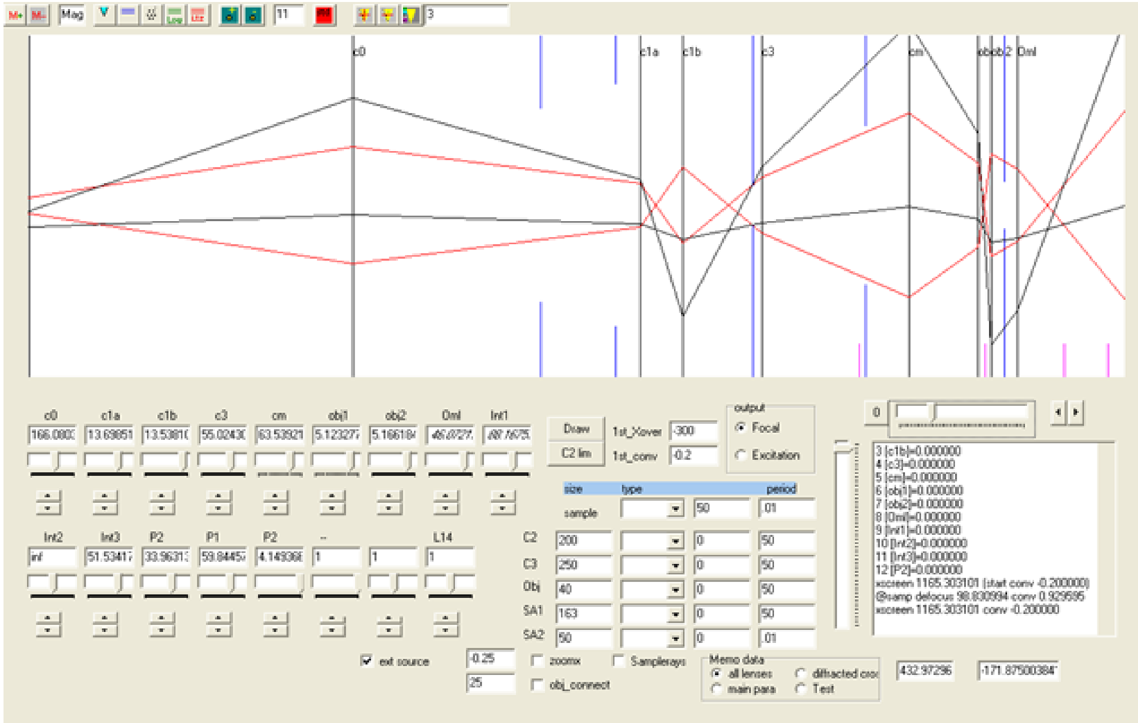

Figure S8: Schematic configuration of lenses where rays departing from two different points in the source end up in the same area in the CA plane, but in different points in the sample plane.

and the other close to the plane wave condition. This condition can be achieved by using the membrane in the PELM stage as a limiting aperture. The thin membrane used for PELM is electron transparent only within a window of typically 100 μm $\times$ 100 μm, effectively functioning as an aperture. Moreover, additional features, such as smaller circular apertures, can be introduced through techniques like thermal evaporation or focused ion beam (FIB) nanofabrication, enabling finer control over the interaction region.

As an example of how the tandem of the two apertures can work, we show now the case of the CA and the pre-CL PELM. As visible in Fig. S10 below such double aperture system would be able to cut all rays originating from an off-center part of the tip and thus propagating within the column several microns away from the optical axis. These are the most incoherent and aberrated rays of the beam. The way for this to work would be to slightly change the excitation of C1b so that the crossover is set closer to the CA aperture. We can reach smaller and smaller effective size at the cost of the intensity, although requiring

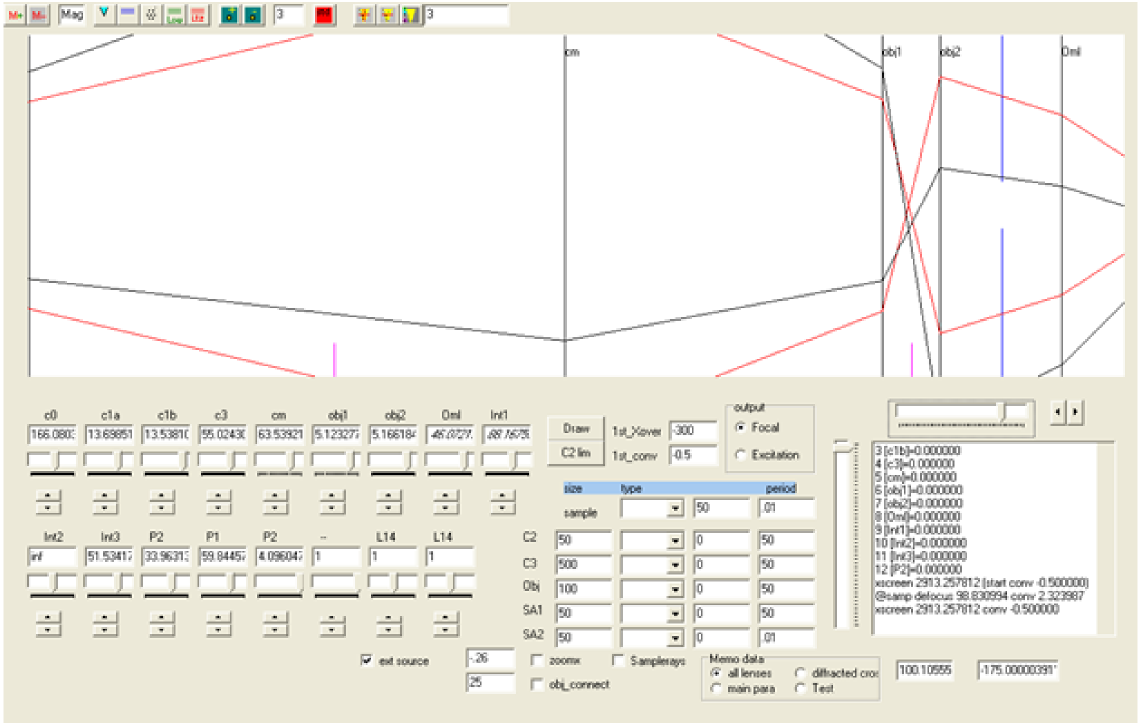

Figure S9: Close-up next to the sample plane of the ray-diagram shown in Fig. S8

smaller and smaller CA ad pre-CL PELM aperture. It is worth noticing that even if the beam at the pre-CL PELM aperture is strongly incoherent, the further effect of the CA is able to remove most of the incoherent part of the source rays and rebuild the coherence afterward (at the sample plane).

The HXA is located after the CA and is hosting the PELM in the post-CL PELM configuration. As shown in Fig. S11, also for this case we can show that we can obtain an effective removal of the rays emitted laterally from the tip, and therefore a tunable increase of the coherence. The main difference here appears to be the size of the beam at the HXA plane, which appears to be a factor 2-3 larger than in the pre-CL PELM position. This feature can be of some advantage in light-mediated beam shaping when the numerical aperture of the illumination system remains low. The exact magnification however would depend on the details of the excitation of the lenses.

The pre-CL PELM has many advantages in terms of technical implementation, as it is easy to construct a spatial interaction point and inspect it. Moreover, the additional section

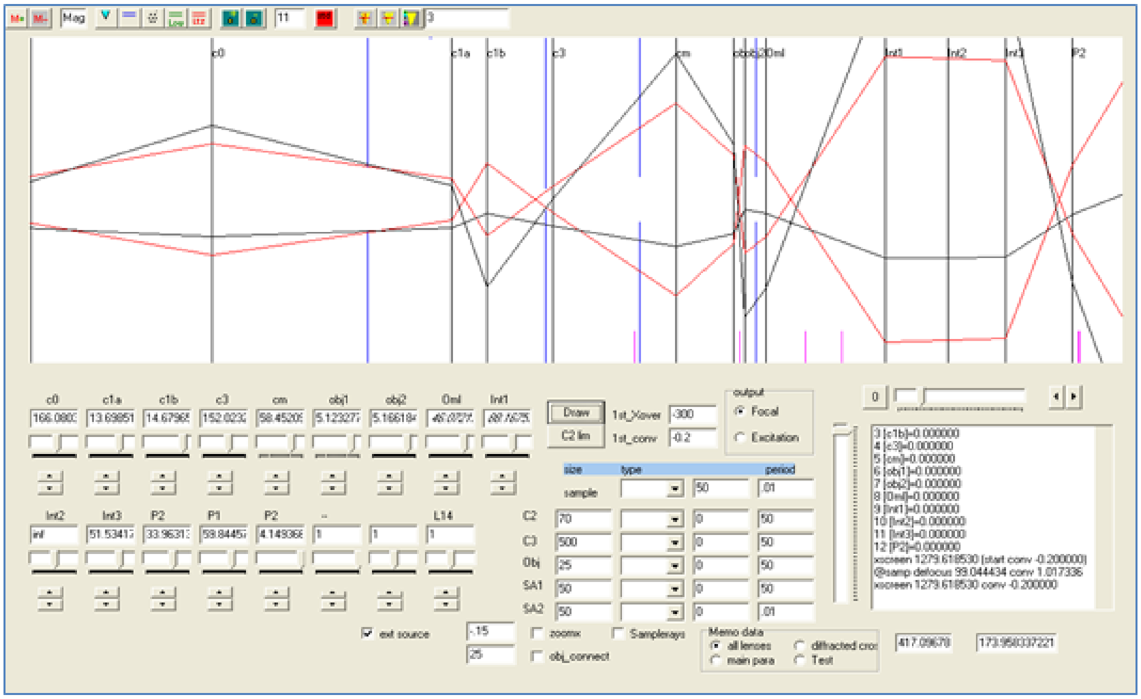

Figure S10: Schematic ray-diagram of the lens system with a pre-CL PELM working in tandem with a condenser aperture CA for post-selection retrieval of electron beam coherence.

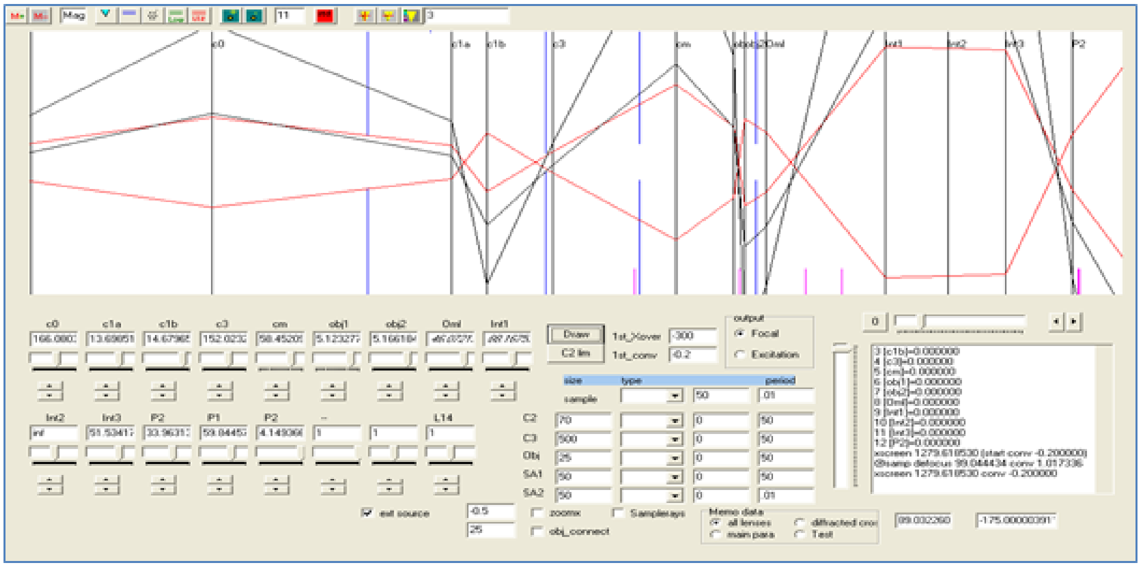

Figure S11: Schematic ray-diagram of the lens system with a post-CL PELM (at the HXA plane) working in tandem with a condenser aperture CA for tunable electron beam coherence.

allows enough space to be available for all possible configurations and setups. From an electron-optics point of view, coherence of the electron beam can be retrieved when it is used in combination with a condenser aperture. In terms of work flow for experiments, we

would need to align it on an intense although incoherent beam (without CA), and then insert CA aperture before measurements. However, if alignment can be done while keeping the CA inserted the effective result of the screen should be similar to the HXA case. The post-CL PELM at the HXA plane offers a larger beam, so the demand for a high numerical aperture illumination scheme can be relaxed. This is however compensated by the difficult geometry that indeed forces to use a limited numerical aperture.

When considering complementarity with the CA for beam control, the post-CL configuration appears to offer a slight advantage compared to the pre-CL case. Specifically, the rejection of the peripheral parts of the beam seems more effective in the HXA plane, although this may depend on the precise lens excitation settings. Furthermore, since the post-CL PELM is positioned after the CA, the electron beam at this stage is already more coherent. This enhanced coherence simplifies alignment and centering during the setup phase, allowing for easier adjustment of the shaping aperture.

In summary, both configurations—pre-CL and post-CL PELM—demonstrate comparable efficacy. The choice between them depends on the specific details of the PELM design and the experimental requirements, with each offering unique advantages.

References

(S1) Williams, D. B.; Carter, C. B. In *Transmission Electron Microscopy: A Textbook for Materials Science*; Williams, D. B., Carter, C. B., Eds.; Springer US: Boston, MA, 2009; pp 141–171.

(S2) Vanacore, G. M.; Madan, I.; Berruto, G.; Wang, K.; Pomarico, E.; Lamb, R. J.; McGrouther, D.; Kaminer, I.; Barwick, B.; García De Abajo, F. J.; Carbone, F. Attosecond Coherent Control of Free-Electron Wave Functions Using Semi-Infinite Light Fields. *Nature Communications* **2018**, *9*, 2694.

(S3) Madan, I.; Leccese, V.; Mazur, A.; Barantani, F.; LaGrange, T.; Sapozhnik, A.;

Tengdin, P. M.; Gargiulo, S.; Rotunno, E.; Olaya, J.-C.; Kaminer, I.; Grillo, V.; De Abajo, F. J. G.; Carbone, F.; Vanacore, G. M. Ultrafast Transverse Modulation of Free Electrons by Interaction with Shaped Optical Fields. *ACS Photonics* **2022**, *9*, 3215–3224.

(S4) Bucher, T.; Nahari, H.; Herzig Sheinfux, H.; Ruimy, R.; Niedermayr, A.; Dahan, R.; Yan, Q.; Adiv, Y.; Yannai, M.; Chen, J.; Kurman, Y.; Park, S. T.; Masiel, D. J.; Janzen, E.; Edgar, J. H.; Carbone, F.; Bartal, G.; Tsesses, S.; Koppens, F. H. L.; Vanacore, G. M.; Kaminer, I. Coherently Amplified Ultrafast Imaging Using a Free-Electron Interferometer. *Nature Photonics* **2024**, *18*, 809–815.

(S5) Grillo, V.; Rotunno, E. STEM_CELL: A Software Tool for Electron Microscopy: Part I—Simulations. *Ultramicroscopy* **2013**, *125*, 97–111.

(S6) Grillo, V.; Rossi, F. STEM_CELL: A Software Tool for Electron Microscopy. Part 2 Analysis of Crystalline Materials. *Ultramicroscopy* **2013**, *125*, 112–129.
